# Supplementary material for: Ecological trade-offs drive phenotypic and genetic differentiation of Arabidopsis thaliana in Europe
Source: Nat Commun. 2024 Jun 18;15:5185. doi: 10.1038/s41467-024-49267-0 (PMC11189578; doi:10.1038/s41467-024-49267-0)
Supplement: Supplementary file 1 — Supplementary Information [file 41467_2024_49267_MOESM1_ESM.pdf]

Supplementary information for

Ecological trade-offs drive phenotypic and genetic differentiation of *Arabidopsis thaliana* in Europe

Cristina C. Bastias[1,2*], Aurélien Estarague[1,3], Denis Vile[3], Elza Gaignon[1], Cheng-Ruei Lee[4], Moises Exposito-Alonso[5], Cyrille Violle[1], François Vasseur[1]

[1] CEFE, Univ Montpellier, CNRS, EPHE, IRD, Montpellier, France.

[2] Área de Ecología, Facultad de Ciencias, Universidad de Córdoba, Campus de Rabanales, 14071 Córdoba, Spain.

[3] LEPSE, Univ Montpellier, INRAE, Institut Agro Montpellier, Montpellier, France

[4] Institute of Ecology and Evolutionary Biology & Institute of Plant Biology, National Taiwan University, Taipei 10617, Taiwan

[5] Department of Plant Biology, Carnegie Institution for Science, Stanford, CA 94305, USA

These authors equally contributed to this work: Cristina C. Bastias, Aurélien Estarague

These authors jointly supervised this work: François Vasseur, Cyrille Violle.

* Corresponding author:

Cristina C. Bastias.

CEFE, Univ Montpellier, CNRS, EPHE, IRD, Montpellier, France.

Área de Ecología, Facultad de Ciencias, Universidad de Córdoba, Campus de Rabanales, 14071 Córdoba, Spain.

crbasc@gmail.com

The supplementary file includes:

Supplementary Figures

Supplementary Fig. 1: Experimental treatments.
Supplementary Fig. 2: Variation of genotypic mean traits across latitude.
Supplementary Fig. 3: Variation of cross-validation error with different number of groups.
Supplementary Fig. 4: Phenotypic variation across widely-distributed *A. thaliana* genotypes in Europe.
Supplementary Fig. 5: Comparison of null vs. observed values of study mean trait for each geographical group.
Supplementary Fig. 6: Linear correlations between phenotypic traits.
Supplementary Fig. 7: Competition vs. colonization tradeoff within each biogeographical group.
Supplementary Fig. 8: Genetic variance structuration between range margins and central genotypes.
Supplementary Fig. 9: Linear correlation between SNP effects.

Supplementary Fig. 10: Number of SNPs in common among 1% top-SNPs on each trait.
Supplementary Fig. 11: Pairwise F_{STQ} / F_{ST} ratio comparisons.
Supplementary Fig. 12: Comparison of F_{STQ}/F_{ST} between biogeographical groups.
Supplementary Fig 13: Variation of relict haplotypes abundances between groups.

Tables

Supplementary Table 1: Summary of the geographical and genetic characteristics of the 71 genotypes of *Arabidopsis thaliana* used in this study.
Supplementary Table 2: Variation in fecundity, plant height, seed mass, and the plant response to water stress and intraspecific competition between biogeographical groups.
Supplementary Table 3: Broad-sense heritability (H^2) for each trait studied.
Supplementary Table 4: Comparison of F_{STQ}/F_{ST} between biogeographical groups with different cutoff values (0.5%, 2% and 5%) of top-SNPs.

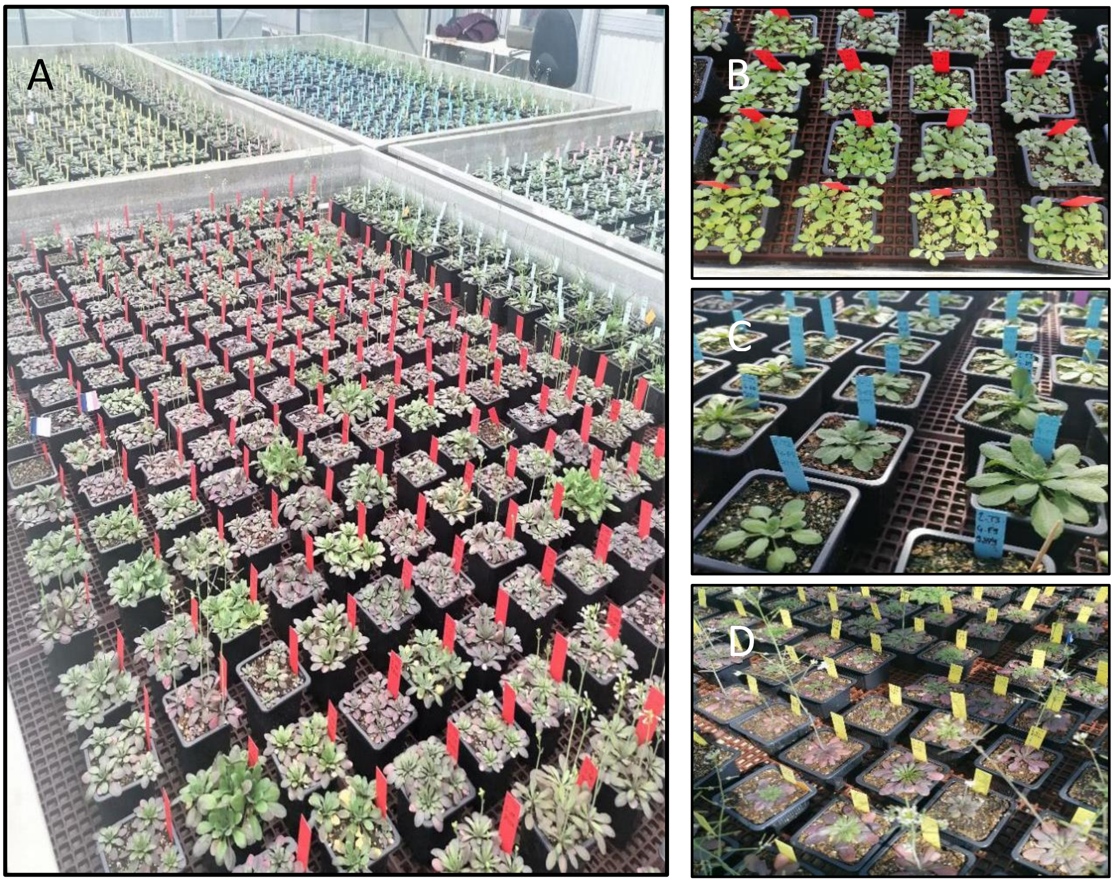

Supplementary Fig. 1: Experimental treatments. (**A**) View of one of the two greenhouse compartments during the experiment; (**B**) Intraspecific competition, i.e. an *A. thaliana* focal plant surrounded by four genotypic neighbors; (**C**) Control environment, i.e. a single plant grown under well watering and without competition, and (**D**) Water stress environment, i.e. a single plant grown without competition but without irrigation during a period of 10-11 days.

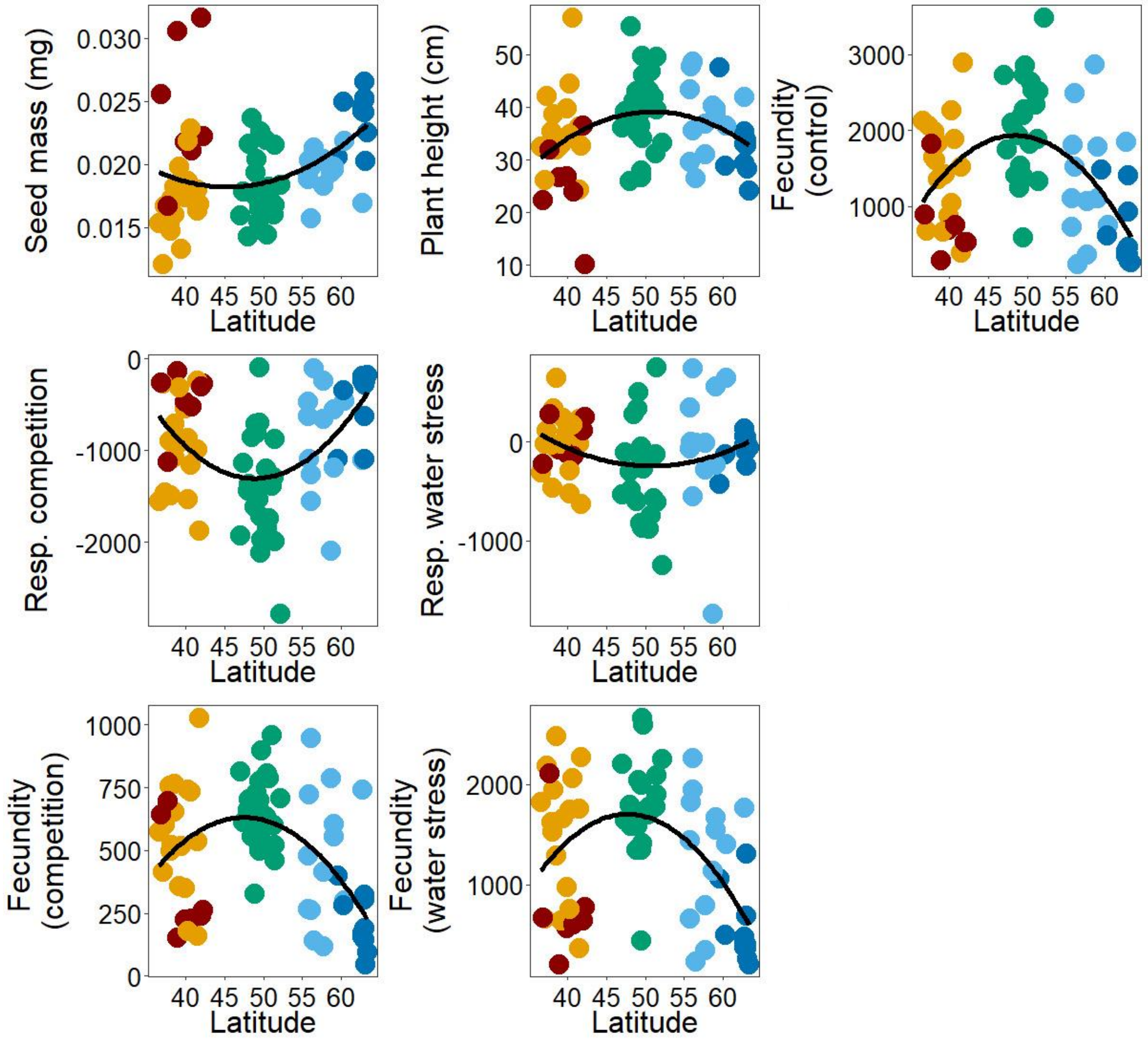

Supplementary Fig. 2: Variation of genotypic mean traits across latitude. Each point represents one genotype categorized within a biogeographical group according to its geographical and genetic origin. Colors represent the biogeographical groups: South relict (dark red), South cosmopolitan (yellow), Center cosmopolitan (green), North cosmopolitan (light blue) and North relict (dark blue).

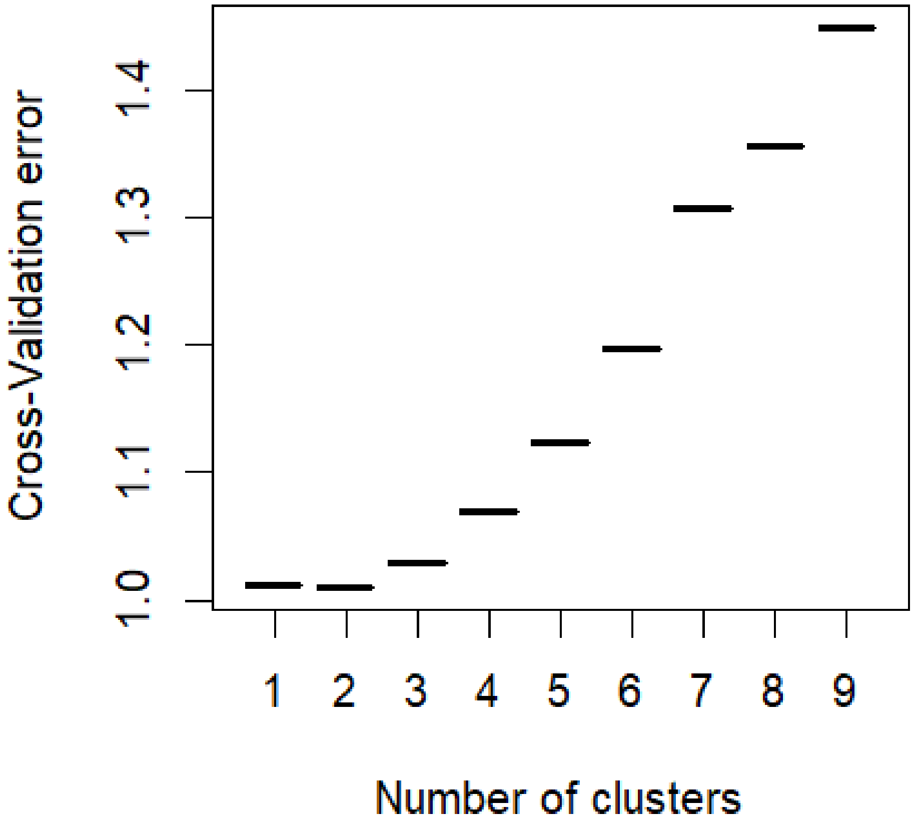

Supplementary Fig. 3: Variation of cross-validation error with different number of groups.

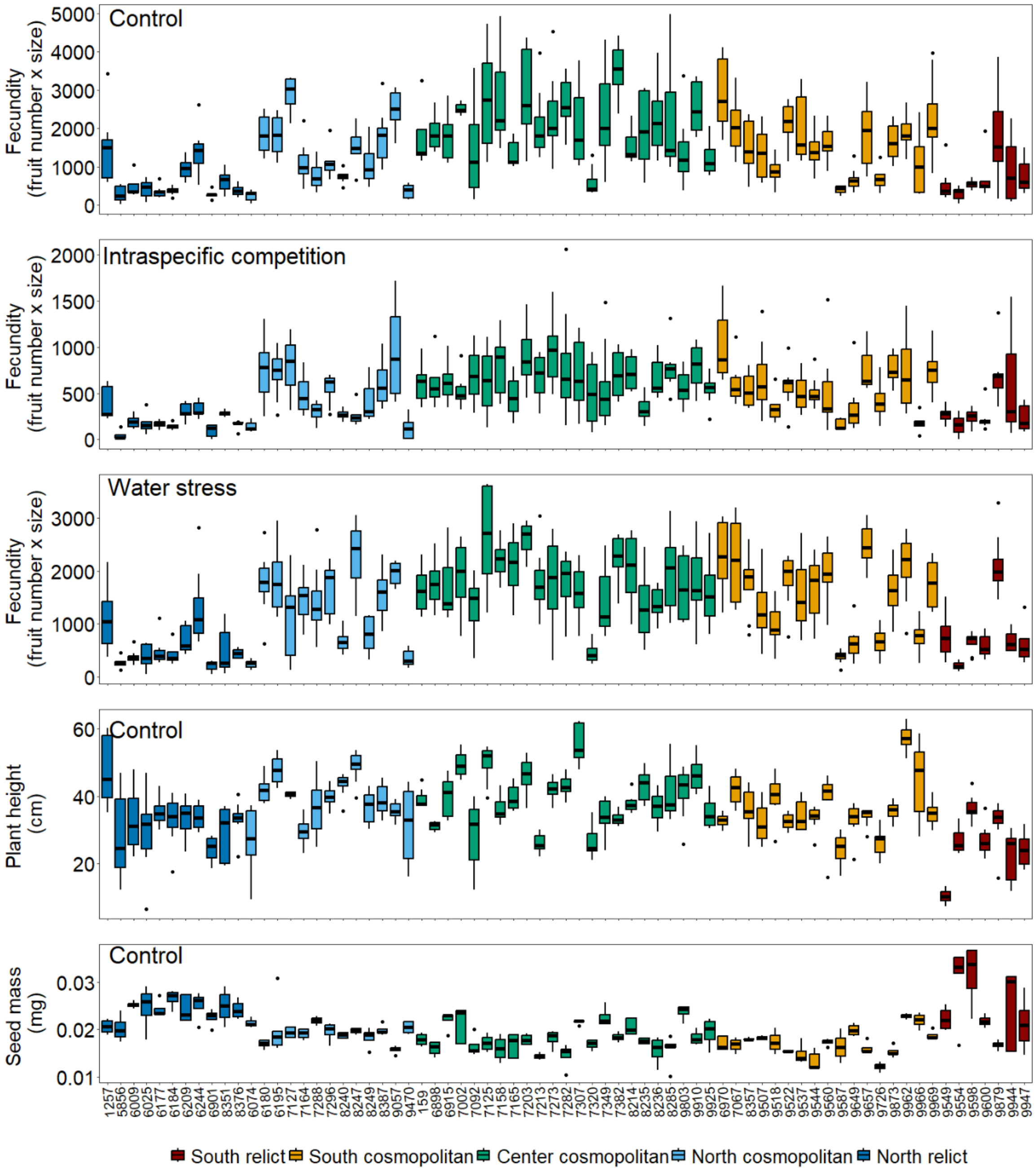

Supplementary Fig. 4: Phenotypic variation across widely-distributed *A. thaliana* genotypes in Europe. Phenotypic variation of fecundity in control, competition and water stress conditions and plant height and seed mass in control conditions measured for 8 individual plants from each natural genotype (In total, *n* = 71 genotypes). Colors represent the biogeographical groups: South relict (dark red), South cosmopolitan (yellow), Center cosmopolitan (green), North cosmopolitan (light blue) and North relict (dark blue). Plot boxes show the minimum, first quartile, median, third quartile and maximum value.

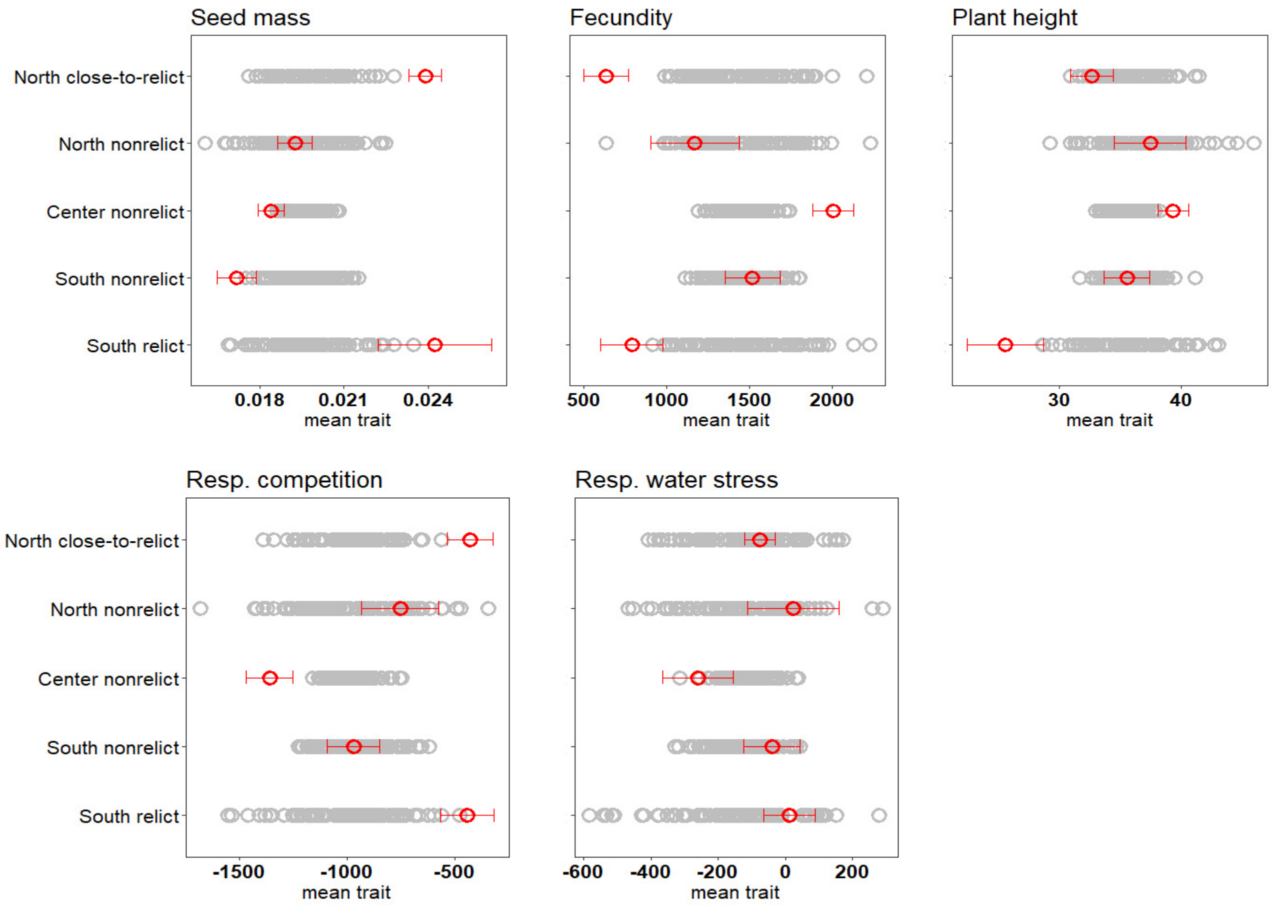

Supplementary Fig. 5: Comparison of null vs. observed values of study mean trait for each geographical group. Red points represent the observed mean trait values and standard errors, and grey ones represent the randomized mean trait values after 100 times shuffling labels of biogeographical groups and keeping the same sample size per group.

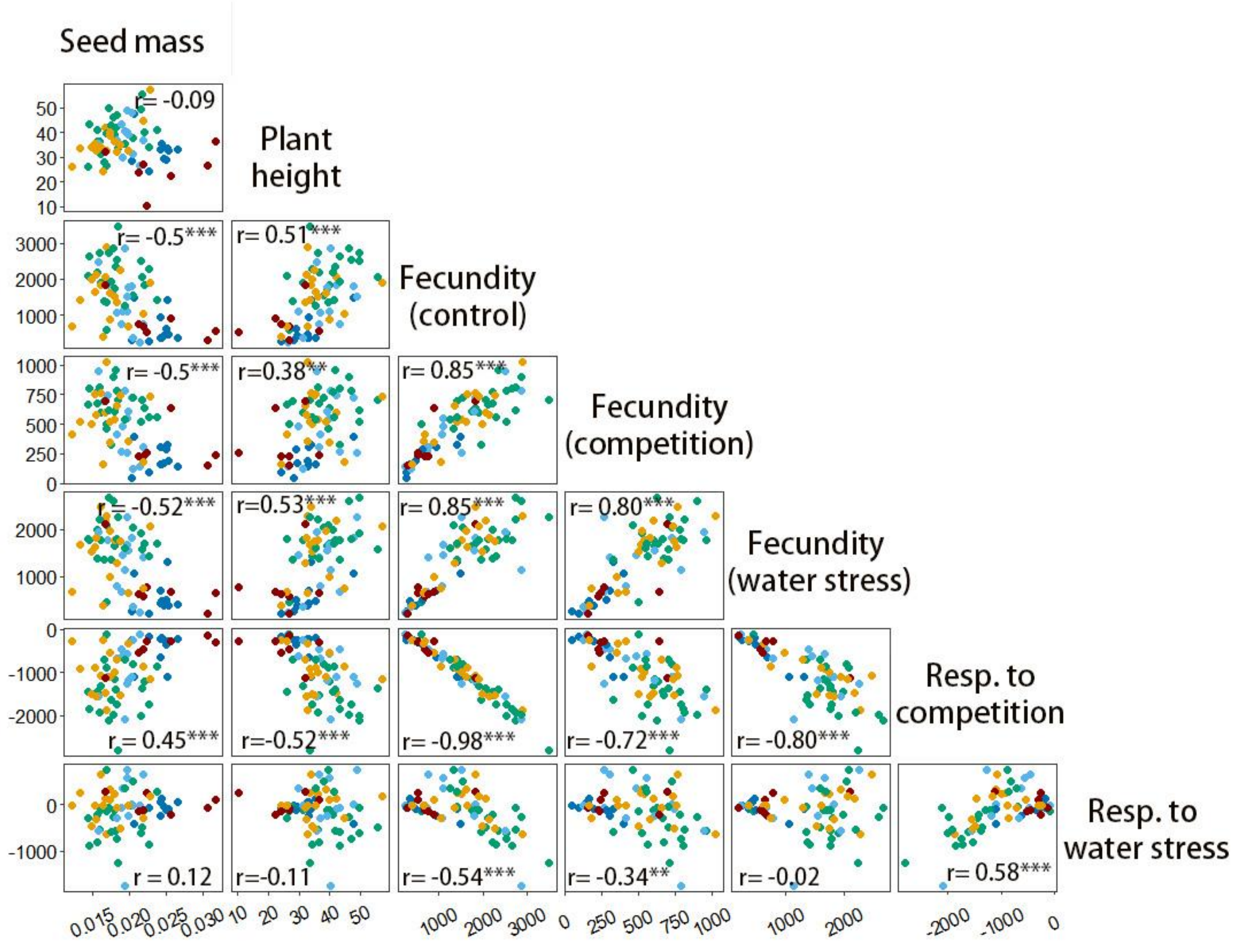

Supplementary Fig. 6: Linear correlations between phenotypic traits. Each point in scatterplot panels represents trait average per genotype. Colors represent the biogeographical groups: South relict (dark red), South cosmopolitan (yellow), Center cosmopolitan (green), North cosmopolitan (light blue) and North relict (dark blue). Pearson's correlation coefficients are * *P* < 0.05, ** *P* < 0.01, *** *P* < 0.001. The exact *P*-values can be seen in 'Source Data file'.

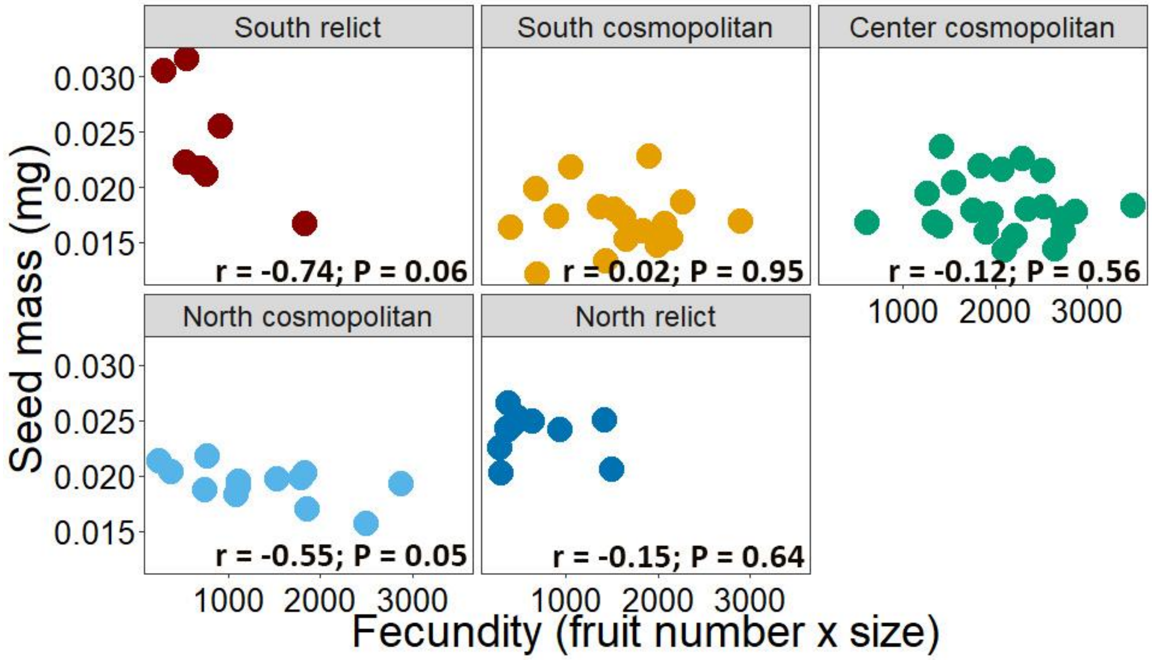

Supplementary Fig. 7: Competition vs. colonization trade-off within each biogeographic group. Mean seed mass estimated from seeds (n = 10-30 air-dried seeds) produced by 4-5 individuals per *A. thaliana* genotype. Fecundity measured as the total number of fruits per genotype multiplied by the average fruit length (n = 8 individual plants per genotype). Each point represents the mean trait value of one *A. thaliana* genotype (n = 7 for South relict; n = 17 for South cosmopolitan; n = 23 for Center cosmopolitan; n = 13 for North cosmopolitan; n = 11 for North relict). Pearson's correlation coefficient (*r*) and statistical significance (*P*) are shown.

A

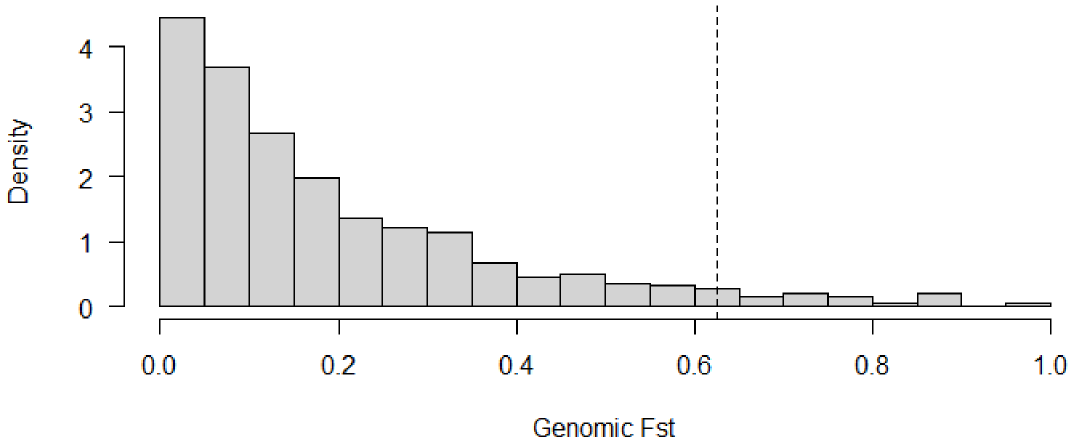

B

traits	Qst	1st Qu.	3rd Qu.	Relative quantile in Fst distribution (%)
seed mass	0.58	0.52	0.64	93,5
plant height	0.35	0.27	0.43	82,2
fecundity	0.47	0.41	0.52	89,3
resp. to competition	0.37	0.31	0.43	84,4
resp. to water stress	0.06	0.01	0.1	25,3

Supplementary Fig. 8: Genetic variance structuration between range margins and central genotypes. (A) F_{ST} distribution of 897 541 SNPs outside genes along the genome. Quantile at 95% of the distribution is shown by a vertical line. **(B)** Estimation of Q_{ST} of traits and their quartiles. The relative quantile of Q_{ST} in the genomic F_{ST} distribution was estimated via bootstrap. The higher was the relative quantiles, the higher was the probability of Q_{ST} values to be the result of selective processes.

A) At the whole-genome level

SNPs related to	Seed mass	Fecundity	Plant height	Fecundity resp. to competition
Seed mass				
Fecundity	**-0.009***			
Plant height	**0.010***	**0.076***		
Fecundity resp. to competition	**0.007***	**-0.278***	**-0.082***	
Fecundity resp. to water stress	0.001	**-0.071***	**-0.004**	**0.087***

SNPs related to

B) At the 1% top-SNPs

SNPs related to	Seed mass	Fecundity	Plant height	Fecundity resp. to competition
Seed mass				
Fecundity	-0.148			
Plant height	0.079	0.140		
Fecundity resp. to competition	**0.334***	**-0.318***	**-0.455***	
Fecundity resp. to water stress	0.237	**-0.289***	**-0.303***	**0.346***

SNPs related to

Supplementary Fig. 9: Linear correlation between SNP effects. Pearson's coefficients of correlation between SNP effects at the whole-genome level **(A)** and at 1% top-SNPs **(B)** related to each study trait. Cells are colored according to coefficient value (red: $r < 0$; blue: $r > 0$). *: $P < 0.05$; **: $P < 0.01$; ***: $P < 0.001$. The exact P-values can be seen in 'Source Data file'.

SNPs related to	Seed mass	Fecundity	Plant height	Fecundity resp. to competition
Seed mass				
Fecundity	45			
Plant height	49	65		
Fecundity resp. to competition	38	427	89	
Fecundity resp. to water stress	31	154	57	217

SNPs related to

Supplementary Fig. 10: Number of SNPs in common among 1% top-SNPs on each trait.

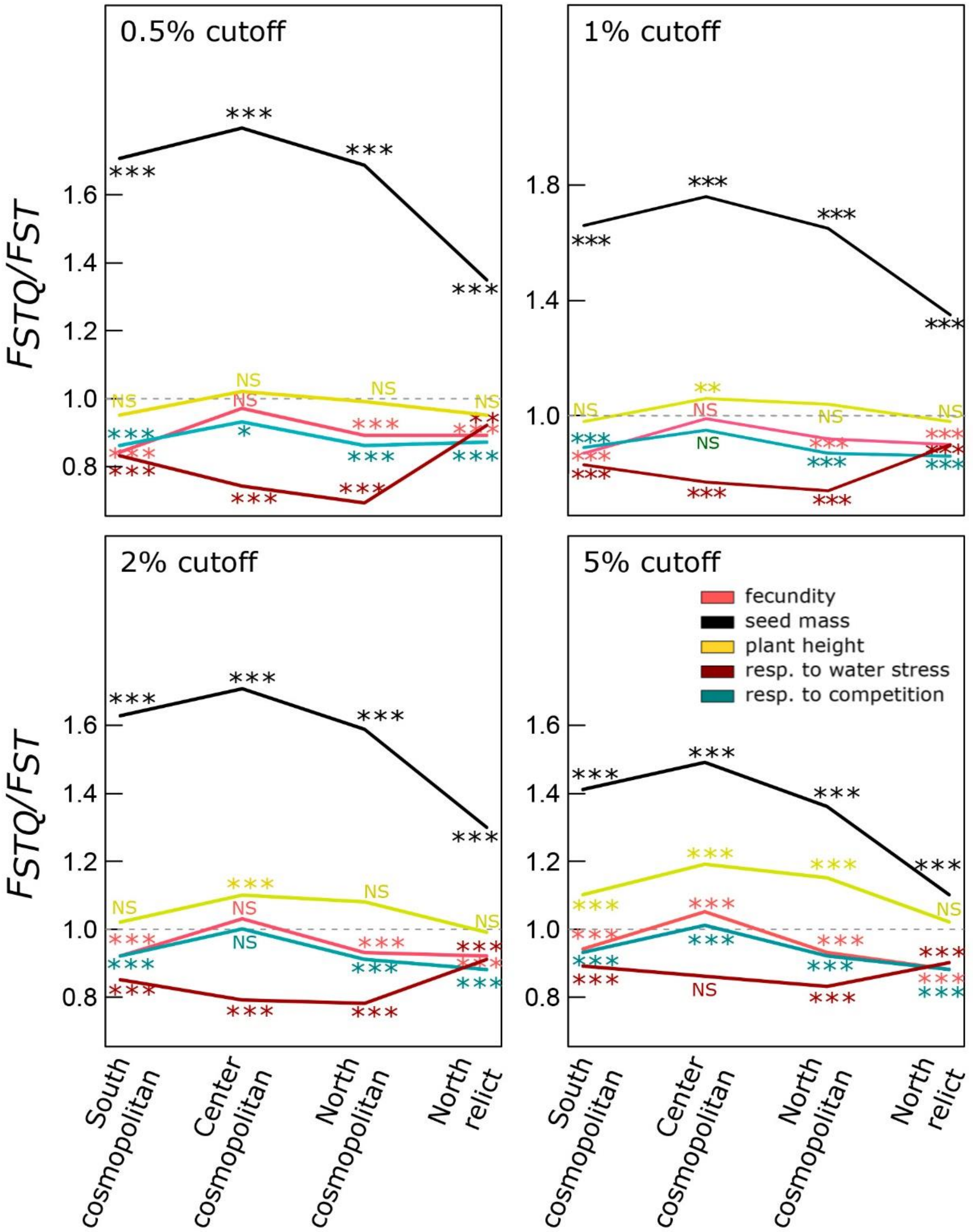

Supplementary Fig. 11: Pairwise F_{STQ} / F_{ST} ratio comparisons. Pairwise F_{STQ} / F_{ST} ratio comparisons between South and North relicts and cosmopolitan groups for every study trait using four different top-SNPs cutoff values, i.e. 0.5%, 1%, 2%, and 5%. The significance of F_{STQ} / F_{ST} ratio was tested with a simple linear model through comparing F_{ST} in non-coding regions versus F_{ST} in top-SNPs of a trait for each cutoff value. NS.: not significant; #: P < 0.1; *: $P < 0.05$; **: $P < 0.01$; ***: $P < 0.001$. The exact P-values can be seen in 'Source Data file'.

F_{STQ}/F_{ST} seed mass				
South relict				
**1.66*** **	South cosmop.			
**1.76*** **	**1.13*** **	Center cosmop.		
**1.65*** **	**1.24*** **	1.57*	North cosmop.	
**1.35*** **	**1.47*** **	**1.51*** **	**1.56*** **	North relict

F_{STQ}/F_{ST} fecundity				
South relict				
**0.87*** **	South cosmop.			
0.99	1.04	Center cosmop.		
**0.92*** **	0.97	**3.56*** **	North cosmop.	
**0.90*** **	1.03	**1.19*** **	1.00	North relict

F_{STQ}/F_{ST} plant height				
South relict				
0.98	South cosmop.			
**1.06** **	0.99	Center cosmop.		
1.04	**0.95* **	1.27	North cosmop.	
0.98	1.06	1.07**	1.06*	North relict

F_{STQ}/F_{ST} resp. to competition				
South relict				
**0.89*** **	South cosmop.			
**0.95**	**1.19*** **	Center cosmop.		
**0.87*** **	1.01	**4.27*** **	North cosmop.	
**0.85*** **	**1.04**	**1.19*** **	1.00	North relict

F_{STQ}/F_{ST} resp. to water stress				
South relict				
**0.83*** **	South cosmop.			
**0.77*** **	**1.00**	Center cosmop.		
**0.74*** **	**0.93* **	1.42	North cosmop.	
**0.90*** **	**0.93*** **	**0.90*** **	**0.85*** **	North relict

Supplementary Fig. 12: Comparison of F_{STQ}/F_{ST} between biogeographical groups. Genetic differentiation in F_{STQ}/F_{ST} calculated from 1% top-SNPs related to each study trait between all biogeographical groups (South and North relicts and South, Center and North cosmopolitan). * $P < 0.05$, ** $P < 0.01$, *** $P < 0.001$. The exact P-values can be seen in 'Source Data file'.

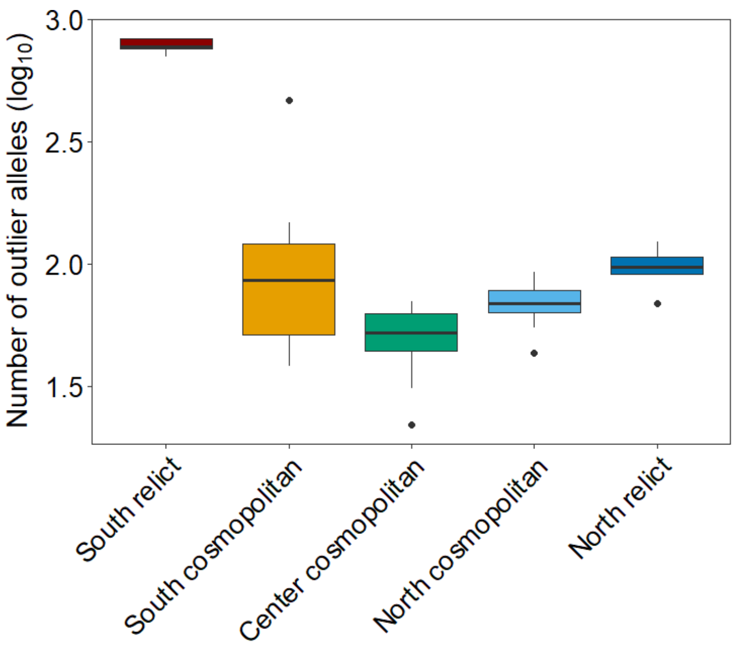

Supplementary Fig. 13: Variation of relict haplotypes abundances between groups. Number of outlier haplotypes (log_{10}), i.e. genomic proportion of relict ancestry in short windows (10 kb) across the genome for each *A. thaliana* natural genotype (*n* = 11 for North relict, *n* = 13 for North cosmopolitan, *n* = 23 for Center cosmopolitan, *n* = 17 for South cosmopolitan and *n* = 7 for South relict group) categorized in each biogeographical group. Plot boxes show the minimum, first quartile, median, third quartile and maximum value.

Supplementary Table 1: Summary of the geographical, and genetic characteristics of the 71 genotypes of *Arabidopsis thaliana* used in this study. Seeds were supplied from the Eurasian Nottingham Arabidopsis Stock Centre (NASC) and the Arabidopsis Biological Resource Center (ABRC). [1]Genotype classification used in this study according to geographical and genetic clustering. [2]Genetic classifications according to the 1001 Genomes project (http://1001genomes.org/).

Accession ID	Name	Country	Biogeographical group[1]	Genetic cluster[2]	Latitude	Longitude
**159**	MAR2-3	France	Center cosmopolitan	Western Europe	47.35	3.933
**1257**	Tos-95-393	Sweden	North relict	North Sweden	59.433	17.016
**5856**	Dör-10	Sweden	North relict	North Sweden	63.016	17.491
**6009**	Eden-1	Sweden	North relict	North Sweden	62.877	18.177
**6025**	Gro-3	Sweden	North relict	North Sweden	62.643	17.733
**6074**	Ör-1	Sweden	North cosmopolitan	South Sweden	56.457	16.130
**6177**	TÄL 03	Sweden	North relict	North Sweden	62.632	17.69
**6180**	TÄL 07	Sweden	North cosmopolitan	Germany	62.632	17.690
**6184**	TBÖ 01	Sweden	North relict	North Sweden	62.889	18.452
**6195**	TDr-9	Sweden	North cosmopolitan	South Sweden	55.770	14.134
**6209**	TEDEN 02	Sweden	North relict	North Sweden	62.883	18.184
**6244**	TRÄ 01	Sweden	North relict	North Sweden	62.916	18.472
**6898**	An-1	Belgium	Center cosmopolitan	Admixed	51.216	4.4
**6901**	Bil-7	Sweden	North relict	North Sweden	63.324	18.484
**6915**	Ei-2	Germany	Center cosmopolitan	Germany	50.3	6.3
**6970**	Ts-1	Spain	South cosmopolitan	Spain	41.719	2.9305
**7002**	Baa-1	Netherlands	Center cosmopolitan	Germany	51.333	6.1
**7067**	Ct-1	Italy	South cosmopolitan	Central Europe	37.300	15
**7092**	Com-1	France	Center cosmopolitan	Western Europe	49.416	2.823
**7125**	Er-0	Germany	Center cosmopolitan	Germany	49.595	11.008
**7127**	Est	Estonia	North cosmopolitan	Admixed	58.665	24.987
**7158**	Gr-5	Austria	Center cosmopolitan	Central Europe	47	15.5
**7164**	Hau-0	Denmark	North cosmopolitan	South Sweden	55.675	12.568
**7165**	Hn-0	Germany	Center cosmopolitan	Germany	51.347	8.288
**7203**	Krot-0	Germany	Center cosmopolitan	Central Europe	49.631	11.572
**7213**	Ler-0	Germany	Center cosmopolitan	Admixed	47.984	10.871
**7273**	No-0	Germany	Center cosmopolitan	Central Europe	51.058	13.299
**7282**	Or-0	Germany	Center cosmopolitan	Germany	50.382	8.011
**7288**	Oy-0	Norway	North cosmopolitan	Admixed	60.385	6.193
**7296**	Petergof	Rusia	North cosmopolitan	Central Europe	59	29
**7307**	Pn-0	France	Center cosmopolitan	Western Europe	48.065	-2.965
**7320**	Rou-0	France	Center cosmopolitan	Western Europe	49.442	1.098
**7349**	Ta-0	Czech Republic	Center cosmopolitan	Central Europe	49.5	14.5
**7382**	Utrecht	Netherlands	Center cosmopolitan	Admixed	52.091	5.114
**8214**	Gy-0	France	Center cosmopolitan	Western Europe	49	2

**8235**	Hod	Czech Republic	Center cosmopolitan	Central Europe	48.8	17.1
**8236**	HSm	Czech Republic	Center cosmopolitan	Central Europe	49.33	15.76
**8240**	Kulturen-1	Sweden	North cosmopolitan	South Sweden	55.705	13.196
**8247**	San-2	Sweden	North cosmopolitan	South Sweden	56.07	13.74
**8249**	Vimmerby	Sweden	North cosmopolitan	South Sweden	57.7	15.8
**8285**	DraIII-1	Czech Republic	Center cosmopolitan	Central Europe	49.411	16.281
**8351**	Ost-0	Sweden	North relict	North Sweden	60.25	18.37
**8357**	Pla-0	Spain	South cosmopolitan	Spain	41.5	2.25
**8376**	Sanna-2	Sweden	North relict	North Sweden	62.69	18
**8387**	St-0	Sweden	North cosmopolitan	Germany	59	18
**9057**	Vinslöv	Sweden	North cosmopolitan	South Sweden	56.1	13.916
**9470**	Tur-4	Sweden	North cosmopolitan	South Sweden	57.651	14.804
**9507**	IP-Coa-0	Portugal	South cosmopolitan	Spain	38.45	-7.5
**9518**	IP-Alm-0	Spain	South cosmopolitan	Spain	39.88	-0.36
**9522**	IP-Bea-0	Spain	South cosmopolitan	Spain	36.52	-5.27
**9537**	IP-Cum-1	Spain	South cosmopolitan	Spain	38.07	-6.66
**9544**	IP-Gua-1	Spain	South cosmopolitan	Spain	39.4	-5.33
**9549**	IP-Hum-2	Spain	South relict	Relict	42.23	-3.69
**9554**	IP-Lso-0	Spain	South relict	Relict	38.86	-3.16
**9560**	IP-Mot-0	Spain	South cosmopolitan	Spain	38.19	-6.24
**9587**	IP-Tdc-0	Spain	South cosmopolitan	Spain	41.5	-1.88
**9598**	IP-Vim-0	Spain	South relict	Relict	41.88	-6.51
**9600**	IP-Vis-0	Spain	South relict	Relict	39.85	-6.04
**9649**	Bivio-1	Italy	South cosmopolitan	Italy/Balkan/Caucasus	39.13	16.17
**9657**	Melic-1	Italy	South cosmopolitan	Italy/Balkan/Caucasus	38.45	16.04
**9726**	Faneronemi-3	Greece	South cosmopolitan	Italy/Balkan/Caucasus	37.07	22.04
**9803**	Muh-2	Germany	Center cosmopolitan	Central Europe	48.42	8.76
**9873**	IP-Ndc-0	Spain	South cosmopolitan	Spain	37.94	-5.45
**9879**	IP-Per-0	Spain	South relict	Relict	37.6	-1.12
**9910**	BRI-2	France	Center cosmopolitan	Western Europe	50.68	3.52
**9925**	RUM-20	France	Center cosmopolitan	Western Europe	48.91	4.52
**9944**	Don-0	Spain	South relict	Relict	36.83	-6.36
**9947**	Ped-0	Spain	South relict	Relict	40.74	-3.9
**9962**	Galdo-1	Italy	South cosmopolitan	Italy/Balkan/Caucasus	40.57	15.32
**9966**	Monte-1	Italy	South cosmopolitan	Italy/Balkan/Caucasus	40.28	15.65
**9969**	Valsi-1	Italy	South cosmopolitan	Italy/Balkan/Caucasus	40.18	16.45

Supplementary Table 2: Variation in fecundity, plant height, seed mass, and the plant response to water stress and intraspecific competition between biogeographical groups. Outputs of the linear mixed models, showing R^2_m: R-squared for fixed factors -marginal-R^2-; and R^2_c: R-squared including random factors -conditional R^2-. Data shown are the *F*-values and *P*-values, the degrees of freedom (*df*) and the statistical significance level of each model using Type II tests.

	Biogeographical group *(F-value)*	*df*	*P-value*	R^2_m	R^2_c
Fecundity in control (Number of fruits x mean fruit lengths)	**53.27**	4	7.46e-11	0.29	0.68
Fecundity in competition	**50.7**	4	2.57e-10	0.23	0.56
Fecundity in water stress	**55.17**	4	1.26e-10	0.30	0.75
Max. plant height (cm)	**22.87**	4	1.34e-4	0.17	0.66
Seed mass (mg)	**60.19**	4	2.67e-12	0.34	0.64
Response to water stress	1.45	4	0.224	0.07	-
Response to competition	**9.56**	4	3.64e-06	0.35	-

Supplementary Table 3: Broad-sense heritability (H^2) for each study trait. H^2 was calculated at the proportion of genotypic variance (σ^2_G) over the total variance (σ^2_G + σ^2_E).

	H^2
Fecundity in control *(number of fruits x mean fruit lengths)*	0.55
Fecundity in competition	0.40
Fecundity in water stress	0.61
Max. plant height (cm)	0.62
Seed mass (mg)	0.63

Supplementary Table 4: Comparison of F_{STQ}/F_{ST} between biogeographical groups with different cutoff values (0.5%, 2% and 5%) of top-SNPs. * $P < 0.05$, ** $P < 0.01$, *** $P < 0.001$. The exact *P*-values can be seen in 'Source Data file'.

F_{STQ}/F_{ST} seed mass			
**South relict**			
0.5% top-SNPs	2% top-SNPs	5% top-SNPs	
**1.71*** **	**1.63*** **	**1.41*** **	South cosmopolitan
**1.80*** **	**1.71*** **	**1.48*** **	Center cosmopolitan
**1.69*** **	**1.59*** **	1.36***	North cosmopolitan
**1.35*** **	**1.30*** **	**1.11*** **	North relict

F_{STQ}/F_{ST} fecundity			
**South relict**			
0.5% top-SNPs	2% top-SNPs	5% top-SNPs	
**0.84*** **	**0.92*** **	**0.94*** **	South cosmopolitan
0.98	1.03	**1.05*** **	Center cosmopolitan
**0.88*** **	**0.92*** **	**0.93*** **	North cosmopolitan
**0.89*** **	**0.93*** **	**0.88*** **	North relict

F_{STQ}/F_{ST} plant height			
**South relict**			
0.5% top-SNPs	2% top-SNPs	5% top-SNPs	
0.95	1.01	**1.10*** **	South cosmopolitan
1.02	**1.11*** **	**1.19*** **	Center cosmopolitan
**0.99* **	1.08***	1.15	North cosmopolitan
0.95	0.99	**1.05*** **	North relict

F_{STQ}/F_{ST} resp. to competition			
**South relict**			
0.5% top-SNPs	2% top-SNPs	5% top-SNPs	
**0.86*** **	0.92	**0.93*** **	South cosmopolitan
**0.93* **	**1.00*** **	**1.01**	Center cosmopolitan
**0.86*** **	0.91***	**0.92*** **	North cosmopolitan
**0.87*** **	0.88***	**0.87*** **	North relict

F_{STQ}/F_{ST} resp. to water stress			
**South relict**			
0.5% top-SNPs	2% top-SNPs	5% top-SNPs	
**0.83*** **	**0.85*** **	**0.89*** **	South cosmopolitan
**0.74*** **	0.79***	**0.86*** **	Center cosmopolitan
**0.69*** **	**0.78*** **	**0.83*** **	North cosmopolitan
**0.92** **	0.91***	**0.90*** **	North relict
