# Supplementary material for: Ecological trade-offs drive phenotypic and genetic differentiation of Arabidopsis thaliana in Europe
Source: Nat Commun. 2024 Jun 18;15:5185. doi: 10.1038/s41467-024-49267-0 (PMC11189578; doi:10.1038/s41467-024-49267-0)
Supplement: Supplementary file 3 — Reporting Summary [file 41467_2024_49267_MOESM3_ESM.pdf]

nature portfolio

Corresponding author(s): Cristina C. Bastias

Last updated by author(s): Apr 15, 2024

Reporting Summary

Nature Portfolio wishes to improve the reproducibility of the work that we publish. This form provides structure for consistency and transparency in reporting. For further information on Nature Portfolio policies, see our Editorial Policies and the Editorial Policy Checklist.

Statistics

For all statistical analyses, confirm that the following items are present in the figure legend, table legend, main text, or Methods section.

n/a	Confirmed	
☐	☒	The exact sample size (*n*) for each experimental group/condition, given as a discrete number and unit of measurement
☐	☒	A statement on whether measurements were taken from distinct samples or whether the same sample was measured repeatedly
☐	☒	The statistical test(s) used AND whether they are one- or two-sided<br>*Only common tests should be described solely by name; describe more complex techniques in the Methods section.*
☐	☒	A description of all covariates tested
☐	☒	A description of any assumptions or corrections, such as tests of normality and adjustment for multiple comparisons
☐	☒	A full description of the statistical parameters including central tendency (e.g. means) or other basic estimates (e.g. regression coefficient) AND variation (e.g. standard deviation) or associated estimates of uncertainty (e.g. confidence intervals)
☐	☒	For null hypothesis testing, the test statistic (e.g. *F*, *t*, *r*) with confidence intervals, effect sizes, degrees of freedom and *P* value noted<br>*Give P values as exact values whenever suitable.*
☐	☒	For Bayesian analysis, information on the choice of priors and Markov chain Monte Carlo settings
☒	☐	For hierarchical and complex designs, identification of the appropriate level for tests and full reporting of outcomes
☐	☒	Estimates of effect sizes (e.g. Cohen's *d*, Pearson's *r*), indicating how they were calculated

Our web collection on statistics for biologists contains articles on many of the points above.

Software and code

Policy information about availability of computer code

Data collection	Phenotypic data was obtained through greenhouse measurements on study accessions. We harvested plants as each one reached maturity, i.e. at plant senescence when fruits started to dry (from mid-April to mid-August 2020), to avoid bias in resource allocation in vegetative or reproductive organs between late and early flowering plants, and then performed a set of phenotypic measurements. For each focal individual, we measured the maximum reproductive height (cm) from the rosette base to the apex of the longest flowering stem. We counted the total number of fruits produced for each focal individual and measured the average fruit length from four fruits chosen randomly along the main inflorescence. We then calculated plant fecundity by multiplying the total number of fruits at whole-plant level by the average fruit length to generate an estimate of the total number of seeds per plant. We measured seed mass (BALCO MC5, mg) of focal individuals (n = 4-5 per genotype) by weighing air-dried seeds (n = 10-30 per individual) and then dividing the total air-dried weight by the number of seeds in the sample. Finally, we measured the fecundity response to competition or water stress as the absolute difference in the mean fecundity of genotype i in stress treatment (intraspecific competition or water stress) and mean fecundity of genotype i in control conditions. We did not use any particular software for data collection.<br>Genomic data for A. thaliana accessions were obtained from the 1,001 Genomes Project. Moreover, we used the proportion of relict ancestry of each genotype, which has been previously calculated by C-RL, coauthor of this study. With this proportion, we later calculated the 'enrichment in outlier haplotypes (%) for every trait and geographical group.
Data analysis	Phenotypic analyses were performed using linear-mixed models from lme4 R-package. For linear mixed-effects models, we used plant height, seed mass, fecundity in control, competition and water stress conditions as response variables. We used the geographical group as a fixed factor and genotype identity (nested within geographical group) and blocks (nested in table and these, in turn, nested in compartment) as random factors. With the same fixed structure, we ran linear models to quantify differences in genotypic mean responses in fecundity to intraspecific competition and water stress among geographical groups. When the variable geographical group was significant, we contrasted mean phenotypic differences among geographical groups through post hoc Tukey's tests using the emmeans R package. Phenotypic analyses

were performed in R software v. 3.5.1.
For genetic analyses, we firstly used the software ADMIXTURE for genetic clustering. Then, we applied Bayesian sparse linear mixed models (BSLMM) implemented in the software GEMMA to determine which SNPs was related to our studied phenotypic traits.
Later, to test whether phenotypic differentiation among geographical groups departed from neutral processes, we compared the structure of genetic variance of traits among populations (QST) to their neutral genetic differentiation using PLINK software. Secondly, we tested in a multi-trait framework if the divergence of trait values across populations was merely explained by drift or natural selection by using the method of Ovaskainen and colleagues. This analysis was carried out using the software R v. 3.5.1 using RAFM and DRIFTSEL R-packages.
Thirdly, we estimated the the significance of FSTQ / FST ratio for each trait and biogeographical group using the SNPs extracted from BSLMMs. Then we performed t-students tests for FSTQ / FST ratio comparisons between biogeographical groups.
Finally, using the calculation of the genomic proportion of relict ancestry for 10 kb window for each genotype previously quantified by the coauthor CR-L, we firstly plotted the number of outlier haplotypes for each one of our geographical groups and then estimated the proportion of outlier haplotypes among our selection of 1% top-SNPs with the strongest positive and negative effect on each studied trait, and among SNPs randomly sampled in non-coding regions. Then, we estimated the average proportion of outlier haplotypes for these three categories (top-positive, top-negative and non-coding) among all genotypes within each geographical group. The 'enrichment in outlier haplotypes (%)' for every trait and geographical group was calculated as the difference between the proportion in outlier haplotypes in top-SNPs and those in non-coding regions.
About code availability, we have included a explicit section explaining the code availability through a reasonable request.

For manuscripts utilizing custom algorithms or software that are central to the research but not yet described in published literature, software must be made available to editors and reviewers. We strongly encourage code deposition in a community repository (e.g. GitHub). See the Nature Portfolio guidelines for submitting code & software for further information.

Data

Policy information about availability of data

All manuscripts must include a data availability statement. This statement should provide the following information, where applicable:

- Accession codes, unique identifiers, or web links for publicly available datasets
- A description of any restrictions on data availability
- For clinical datasets or third party data, please ensure that the statement adheres to our policy

The mean functional trait and genetic data generated in this study have been deposited and are freely available in the Figshare Digital Repository database under accession code https://doi.org/10.6084/m9.figshare.23807346.v298. Accessions used in this study had been previously described by 1001 Genomes project (http://1001genomes.org/) and their codes can be seen in the Supplementary material file (Supplementary Table 1). Source data are provided as a Source Data file.

Research involving human participants, their data, or biological material

Policy information about studies with human participants or human data. See also policy information about sex, gender (identity/presentation), and sexual orientation and race, ethnicity and racism.

Reporting on sex and gender	Not applicable.
Reporting on race, ethnicity, or other socially relevant groupings	Not applicable.
Population characteristics	Not applicable.
Recruitment	Not applicable.
Ethics oversight	Not applicable.

Note that full information on the approval of the study protocol must also be provided in the manuscript.

Field-specific reporting

Please select the one below that is the best fit for your research. If you are not sure, read the appropriate sections before making your selection.

☐ Life sciences ☐ Behavioural & social sciences ☒ Ecological, evolutionary & environmental sciences

For a reference copy of the document with all sections, see nature.com/documents/nr-reporting-summary-flat.pdf

Ecological, evolutionary & environmental sciences study design

All studies must disclose on these points even when the disclosure is negative.

Study description	Although the initial colonization of Arabidopsis thaliana in Europe by relicts can be traced back to the gradual melting of the ice cap, the success of the cosmopolitan group's expansion across Europe remains enigmatic, as does the preservation of relictual genetic variations solely at the peripheries of its range. In the present study, we investigated three questions: (i) do traits differentiate between A. thaliana populations according to a center-to-margin gradient, with northern lines displaying similar trait variations as their genetic relatives at the opposite southern margin? (ii) does trait variation among geographical and genetic groups relate to

major ecological trade-offs between stress resistance, competition tolerance, and dispersal ability? aAnd (iii) what how does it trait variation account for the maintenance of relictual, genetic variation at the opposite margins of the distribution range? To address these questions, we conducted experiments using 71 natural genotypes from three geographical areas (center, south, and north) within the European range of A. thaliana. We investigated phenotypic and genetic correlations between traits, and explored whether phenotypic differentiation toward the range margins could be explained by adaptive introgressions following hybridization between cosmopolitans and relicts.

Research sample	We firstly performed a greenhouse experiment to test phenotypic differences (fecundity, seed mass, plant height, and stress responses to competition and water stress) among A. thaliana accession groups from different geographical origin and genomic basis (by using results of genetic clustering analyses from genomic data from the 1,001 Genomes project (http://1001genomes.org/). We then calculated the adaptive basis of exhibited phenotypic characters for each A. thaliana group. Finally, we calculated the effect of the genomic proportion of relict ancestry of each A. thaliana accession on each study phenotypic trait.
Sampling strategy	We used a total of 71 natural genotypes of A. thaliana from three geographical areas representative of center, south, and north Europe, and which are separated by natural barriers that partially isolate plant populations. We used the latitude threshold of 45º, which corresponds to the Pyrenees and Alps mountains, to delineate the south area, and the latitude threshold of 55° that separate the Scandinavian peninsula. We excluded genotypes originating from sites with an altitudinal distribution above 1000 m a.s.l. to avoid confounding factors associated to elevation (Supplementary Table 1). All genotypes were included in the initial germplasm of the 1,001 Genomes project (http://1001genomes.org/), and seeds were supplied by the Nottingham Arabidopsis Stock Centre (NASC) and the Arabidopsis Biological Resource Center (ABRC). All accessions were included in the initial germplasm of the 1,001 Genomes project (http://1001genomes.org/44), and seeds were supplied from the Nottingham Arabidopsis Stock Centre (NASC) and the Arabidopsis Biological Resource Center (ABRC).<br>We downloaded full genomic sequences from the 1,001 Genomes Project available and filtered SNPs with a minor allele frequencies (MAF) superior to 5% among the 71 accessions. Genetic clustering was performed with ADMIXTURE after linkage disequilibrium pruning (r2 < 0.1 in a 50 kb window with a step size of 50 SNPs) with PLINK, resulting in 47,213 independent SNPs used for subsequent analyses. A cross-validation for different numbers of clusters (k = 1 to k = 9) showed that the set of studied genotypes was best separated into two groups, (lowest cross-validation error, CV = 1.00). Following the same approach as the 1,001 genomes project, we assigned each genotype to a group if more than 50% of its genome derived from the corresponding cluster. Thus, we classified the 71 initially selected accessions into five biogeographical groups, considering the interplay between geographic origin and genetic clustering (Supplementary Table 1). There was a single group in the center: the 'Center cosmopolitan' group (n = 23); and two groups in the south area ('South cosmopolitan', n = 17, and 'South relict', n = 7) and in the north area ('North cosmopolitan', n = 13, and 'North relict', n = 11).<br>The greenhouse experiment was carried out in two adjacent compartments of a greenhouse, where plants were sown and grown in individual pots (7 x 7 x 6.5 cm) and submitted to three different treatments: control, competition and water stress. In the control treatment (non-stressful conditions), three to eight seeds per pot were sown, and the first germinated plant was kept and grown at pot center under well-watered conditions (regular irrigation every 4-5 days during the experiment). In the intraspecific competition treatment, a focal A. thaliana plant was grown at the pot center surrounded by four individuals of the same genotype, under similar well-watered conditions as the control treatment. In the water stress treatment, the first germinated plant was selected at pot center, i.e. without competing neighbors, and grown in water stress conditions – regular irrigation every 10-11 days along the experiment since plants reached three - four true leaves (Supplementary Fig. 1).<br>We used eight pot replicates of each genotype and treatment, resulting in a total of 71 genotypes x 8 replicates x 3 environments = 1,704 pots (Supplementary Fig. 1).
Data collection	Cristina C. Bastias and Aurelien Estarague performed the plant measurements on greenhouse experiment.
Timing and spatial scale	For the first 40 days from sowing (from end of January to mid-March 2020), we kept all plants in cold temperature (~10 ºC) under well-watered conditions to ensure establishment and vernalization of all accessions (i.e. breaking the genetic suppression of flowering). After the vernalization period, we raised the room temperature (20 ºC day / 15 °C night), and applied the different watering treatments to each plant reached maturity and was harvested (mid-march to mid-August 2020). In this way, we assured that all plants had completed the life cycle regardless differential in flowering timing among genotypes or geographical origin.<br>Plants were equally distributed in the two adjacent compartments, with three large tables each containing one treatment per table and compartment. Each table was divided into four similar blocks, that included one replicate per genotype randomly placed. The tables were rotated within the compartments and turned around themselves every two days to minimize putative microclimate heterogeneity in each compartment.
Data exclusions	From the initial 1,704 individuals, we finally conducted analyses over a total of 1,595 individuals, after discarding plants that did not complete their life cycle at the end of experiment or died during the experiment. In total, 544 plants grew under control, 507 grew under intraspecific competition, and 544 were under water stress conditions. In the competition treatment, we did not consider in our analyses the focal plants with absence of one or more of their four neighbors to avoid potential bias linked to neighboring plant density. Further, we removed 32 individuals due to human errors in plant identification and 23 individuals, which were truly measured but showed extreme values within genotype and treatment after applying the Hampel filter, i.e. the median, plus or minus 3 median absolute deviations.
Reproducibility	Once each plant reached maturity, i.e. when fruits start to dry, we measured the maximum reproductive height (cm) from the rosette base to the apex of the longest flowering stem. We counted the total number of fruits produced for each focal individual and measured the average fruit length from four fruits chosen randomly along the main inflorescence. We then calculated plant fecundity by multiplying the total number of fruits at whole-plant level by the average fruit length to generate an estimate of the total number of seeds per plant. On the other hand, we measured seed mass (BALCO MC5, mg) of focal individuals (n = 4-5 per accession) by weighing air-dried seeds (n = 10-30 per individual) and then dividing the total air-dried weight by the number of seeds in the sample. Finally, we measured the fecundity response to competition or water stress as the absolute difference in the mean fecundity of genotype i in stress treatment (intraspecific competition or water stress) and mean fecundity of genotype i in control conditions. The experiment and treatments were successful.

Randomization	Plants were equally distributed in the two adjacent compartments, with three large tables each and containing one treatment per table and compartment. Each table was divided into four similar blocks, that included one replicate per accession randomly placed. The final position of each plant, associated to a genotype, within each block was determined using RANDBETWEEN function in excel.
Blinding	The data acquisition was carried out by Cristina C. Bastias and Aurelien Estarague, working together with the same criteria. Both investigators were blinded to measurement allocation during data collection.

Did the study involve field work? ☐ Yes ☒ No

Reporting for specific materials, systems and methods

We require information from authors about some types of materials, experimental systems and methods used in many studies. Here, indicate whether each material, system or method listed is relevant to your study. If you are not sure if a list item applies to your research, read the appropriate section before selecting a response.

Materials & experimental systems

n/a	Involved in the study	
☒	☐	Antibodies
☒	☐	Eukaryotic cell lines
☒	☐	Palaeontology and archaeology
☒	☐	Animals and other organisms
☒	☐	Clinical data
☒	☐	Dual use research of concern
☐	☒	Plants

Methods

n/a	Involved in the study	
☒	☐	ChIP-seq
☒	☐	Flow cytometry
☒	☐	MRI-based neuroimaging
